# Disparities in Treatment and Referral After an Opioid Overdose Among Emergency Department Patients

**DOI:** 10.1001/jamanetworkopen.2025.18569

**Published:** 2025-07-02

**Authors:** Siri Shastry, Joseph Carpenter, Alex Krotulski, Jeffrey Brent, Paul Wax, Kim Aldy, Sharan Campleman, Rachel Culbreth, Alyssa Falise, Adrienne Hughes, Robert G. Hendrickson, Alexandra Amaducci, Bryan Judge, Christopher Meaden, Diane P. Calello, Jennie Buchanan, Joshua Shulman, Michael Levine, Evan Schwarz, Alex F. Manini

**Affiliations:** 1 Icahn School of Medicine at Mount Sinai, New York, New York; 2 Emory University School of Medicine, Atlanta, Georgia; 3 Grady Health System, Atlanta, Georgia; 4 Center for Forensic Science Research and Education, Fredric Rieders Family Foundation, Horsham, Pennsylvania; 5 University of Colorado School of Medicine, Aurora; 6 American College of Medical Toxicology, Phoenix, Arizona; 7 University of Texas Southwestern Medical Center, Dallas; 8 Oregon Health and Science University, Portland; 9 Lehigh Valley Health Network/USF Morsani College of Medicine, Allentown, Pennsylvania; 10 Corewell Health, Michigan State University, Grand Rapids; 11 Rutgers New Jersey Medical School, Newark; 12 Denver Health and Hospital Authority, Denver, Colorado; 13 University of Pittsburgh School of Medicine, Pittsburgh, Pennsylvania; 14 University of California Los Angeles; 15 Icahn School of Medicine at Mount Sinai, Center for Research on Emerging Substances, Poisoning, Overdose, and New Discoveries (RESPOND), Department of Emergency Medicine, New York, New York; 16 Elmhurst Hospital Center, New York, New York

## Abstract

**Importance:**

There is a disproportionately high rate of overdose deaths immediately following an emergency department (ED) visit for opioid overdose. Thus, an improved understanding of disparities in ED treatment and referral is vital. Racial and ethnic disparities in access to naloxone and buprenorphine have been described in the outpatient setting but prevalence in the ED setting remains understudied.

**Objective:**

To examine racial and ethnic disparities in treatment referral rates in ED patients with opioid overdose.

**Design, Setting, and Participants:**

This is a secondary analysis of a prospective consecutive cohort from the Toxicology Investigators Consortium (TOXIC) Fentalog Study from September 21, 2020, to November 11, 2023. Ten hospital sites were a part of the TOXIC network and participants included ED patients in aged 18 years or older with opioid overdose. Data were analyzed from December 2022 to March 2025.

**Exposures:**

Patient race, ethnicity, sex, and other demographic and clinical factors of interest.

**Main Outcomes and Measures:**

Study outcomes included the proportion of patients receiving a referral to outpatient addiction care and the proportion receiving a naloxone kit or prescription or buprenorphine prescription at discharge. Descriptive statistics were tabulated, and χ^2^ and multivariable logistic regression analyses were used to evaluate for differences by race, ethnicity, sex, and other demographic and clinical variables.

**Results:**

In this study, 1683 patients met all inclusion criteria (mean [SD] age, 42.5 [14.5] years; 1221 males [72.6%]; 461 females [27.4%]; 447 Black patients [26.6%]; 63 Hispanic patients [4.3%]; 867 White patients [51.5%]). Of the 1683 included patients, 299 (17.8%) received a referral for outpatient treatment, 713 (42.4%) received a naloxone kit or prescription, and 141 (8.4%) received a buprenorphine prescription. Compared with White patients, Black patients had a decreased adjusted odds ratio (aOR) of outpatient treatment referral (aOR, 0.67; 95% CI, 0.47-0.97). Hospital admission was also associated with increased adjusted odds of outpatient treatment referral (aOR, 3.13; 95% CI, 2.34-4.20). Geographic variation was associated with all primary and secondary outcomes.

**Conclusions and Relevance:**

In this study, Black patients were less likely to receive outpatient referrals for OUD. These findings underscore the need for targeted interventions to address racial disparities in ED care for OUD, particularly in enhancing referral processes.

## Key Points

**Question:**

Are there racial and ethnic disparities in treatment referral rates among patients in the emergency department (ED) with opioid overdose?

**Findings:**

In this cohort study of 1683 patients, there was a statistically significant difference in the proportion of Black patients who received an outpatient treatment referral (5.7%) compared with White patients (9.6%).

**Meaning:**

These findings suggest that Black patients presenting to the ED with opioid overdose may be less likely to receive outpatient treatment referrals, underscoring the need for targeted intervention and enhanced referral processes.

## Teaser

This secondary analysis of a cohort study examined racial and ethnic disparities in treatment referral rates in emergency department (ED) patients with overdose.

## Introduction

Despite substantial evidence supporting the safety and efficacy of medications for opioid use disorder (MOUD), including buprenorphine, methadone, and injectable extended-release naltrexone, only 25% of adults who needed treatment for opioid use disorder (OUD) received treatment in 2022.^1^ Many patients access health care exclusively or primarily through the emergency department (ED),^2^ and patients with OUD disproportionately use the ED.^3^ Several studies demonstrated ED-based initiation of MOUD, specifically buprenorphine, is feasible and can effectively link patients to ongoing treatment.^4,5^ According to data from the National Hospital Ambulatory Medical Care Survey, ED use of buprenorphine steadily increased from 2002 to 2017; however, this dataset is limited by its inability to determine the reason for the visit (eg, opioid overdose, injection-related infection).^6^ Furthermore, gaps in care, particularly with respect to racial disparities in care, remain physicians’ comfort initiating OUD treatment and ability to arrange postdischarge care.^6,7,8^

Patients seen in the ED for opioid overdose are a particularly vulnerable group. The 1-year mortality rate after an ED visit for opioid overdose is approximately 5%, with a dramatic number of deaths observed in the first days after the encounter.^9,10^ Therefore, these visits represent a crucial opportunity to start evidence-based treatment with buprenorphine or other MOUD and to provide naloxone to treat future overdose. Each of these interventions have the capacity to dramatically decrease mortality following a nonfatal overdose.^11^ One study using commercial insurance claims indicated that only about 16% of patients received MOUD in the 90 days following a nonfatal overdose, with Black patients, non-Hispanic White patients, and women all less likely to receive care.^12^ Another large national study using claims data found that just 8.5% of patients received a prescription for buprenorphine and 7.5% a prescription for naloxone in the 30 days following an ED visit for opioid overdose.^13^

By using insurance databases and claims data, each of these studies underrepresents those who are uninsured or publicly insured, a group encompassing nearly three-fourths of individuals in the US needing OUD treatment.^14^ Extant studies also lack laboratory confirmation of the substances contributing to overdose and ongoing treatment, which may be particularly important with ongoing prevalence of fentanyl and fentanyl analogs, as well as polysubstance use. Furthermore, while the majority of prior observational research in this area has been aimed toward ED-initiated buprenorphine, many patients seen in the ED for opioid overdose are admitted, and there is a need to better understand potential disparities in care between patients discharged from the ED after overdose and those admitted to the hospital for ongoing care. The purpose of this study is to evaluate potential disparities in outpatient treatment linkage, buprenorphine prescribing, and naloxone provision or prescribing after hospital visits for toxicology-confirmed opioid overdose from a network of geographically diverse hospital systems within the US.

## Methods

### Study Design, Setting, and Population

This cohort study was a secondary data analysis of the Toxicology Investigators Consortium (TOXIC) Fentalog Study, an ongoing, prospective, multicenter study across 10 participating health care centers in the US. The cohort for this secondary analysis includes patients entered into the Fentalog Study between September 21, 2020, to November 11, 2023, with completed comprehensive toxicology testing at the time of analysis. This study follows the Strengthening the Reporting of Observational Studies in Epidemiology (STROBE) reporting guideline.^15^

Patients aged 18 years and older were screened for inclusion to the Fentalog Study if they presented to an emergency department with a suspected opioid overdose and waste blood from laboratory specimens sent as part of routine clinical care. Suspected opioid overdose was identified via 1 of 3 methods: (1) chief concern or discharge diagnosis suggesting opioid overdose; (2) receipt of naloxone for overdose reversal in the prehospital setting and/or ED; or (3) self-reported opioid use resulting in signs and symptoms of an overdose on presentation. Exclusion criteria were patients younger than 18 years, patients with co-occurring trauma or burns, or incarcerated persons. Patients with incomplete comprehensive toxicology testing were also excluded from the parent study dataset. Detailed information regarding the inclusion and exclusion criteria can be found in the Figure. The WIRB-Copernicus Group institutional review board (IRB) provided central approval and a waiver of informed consent for the Fentalog Study, and each participating site obtained approval from their local IRB.

**Figure.**
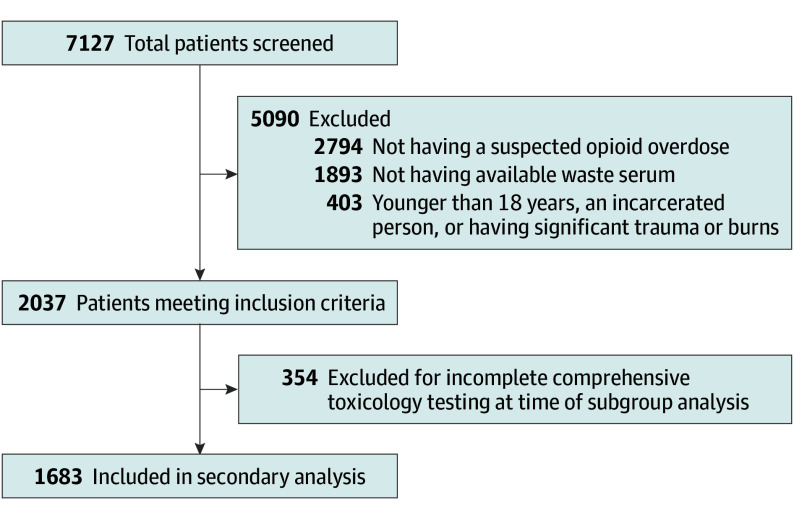
Patient Enrollment and Inclusion

Patients at participating sites were screened and assessed for eligibility by research staff (medical toxicology physicians or fellows, or trained research assistants) using the criteria above. A priori data were collected via medical record review and consisted of demographic variables (eg, age, sex, race, or ethnicity), past medical and psychiatric history, suspected opioid and other substances exposure, clinical characteristics (eg, relevant laboratory data, specific organ toxicity), treatments received (eg, naloxone, nonpharmacological interventions), MOUD initiation, and disposition (ie, discharge, admit, or intensive care unit). Races included Black, White, unknown and other (eg, American Indian or Alaska Native, Asian, Native Hawaiian or Pacific Islander, and mixed or any other race). Ethnicity included Hispanic or non-Hispanic. Patient race or ethnicity and suspected opioid and other substance exposure were patient-reported. Other variables were abstracted from the relevant electronic medical record fields. Data were deidentified and entered into a secure, web-based software platform (Research Electronic Data Capture [ie, RedCAP]) by research staff at each site. Deidentified clinical data was linked to toxicology blood analysis using a unique study identification code. Database quality assurance was maintained by dedicated centralized TOXIC staff in accordance with current best practices, including database logical checks, quality assurance personnel, automated data cleaning, data tracking, encryption, and data abstractor training.

### Outcomes

The primary study outcome was the proportion of patients receiving outpatient treatment referral at discharge. Outpatient treatment referral was operationalized as a scheduled outpatient appointment (verified by medical record review). Secondary outcomes included naloxone at discharge (defined as either receipt of take-home naloxone kit or naloxone prescription), or receipt of a buprenorphine prescription at discharge. These outcomes were monitored through extensive medical record reviews conducted by the sites’ research team.

### Statistical Analysis

Descriptive statistics examining patient demographics and clinical characteristics were tabulated. χ^2^ and Fisher exact tests assessed for significant differences in sociodemographic and health-related characteristics by referrals received upon discharge. Multivariable logistic regression models considered whether sociodemographic characteristics were associated with OUD treatment referrals following adjustment for select demographic and clinical covariates. These included age, clinical site, psychiatric history, overdose intent, and hospital admission.^16,17^ Analyses were conducted using SAS University Edition version 9.4 (SAS Institute) and SPSS version 24 (IBM). Data were analyzed from December 2022 to March 2025. Statistical significance was set at *P* < .05, and all tests were 2-sided.

## Results

During the study period, a total of 7127 patients were screened. Of these patients, 1683 met all study inclusion criteria and had completed toxicology analyte data available at the time of analysis (mean [SD] age, 42.5 [14.5] years; 1221 males [72.6%]; 461 females [27.4%]; 447 Black patients [26.6%]; 63 Hispanic patients [4.3%]; 867 White patients [51.5%]). Full summary of study screening and enrollment can be found in the Figure. The majority of patients had a documented comorbid psychiatric diagnosis (991 patients [59%]). Patient demographics and clinical characteristics are provided in Table 1.

**Table 1.** Demographic and Clinical Characteristics of Included Patients (N = 1683)

Characteristic	Patients, No. (%)
Age, mean (SD), y	42.5 (14.5)
Sex	
Male	1221 (72.6)
Female	461 (27.4)
Race	
Black	447 (26.6)
White	867 (51.5)
Unknown	275 (16.3)
Other^a^	94 (5.6)
Ethnicity	
Hispanic	394 (25.3)
Non-Hispanic	1163 (74.7)
Site	
Atlanta	83 (4.9)
Bethlehem	164 (9.7)
Denver	255 (15.2)
Grand Rapids	116 (6.9)
Los Angeles	79 (4.7)
New York	477 (28.3)
Newark	87 (5.2)
Pittsburgh	160 (9.5)
Portland	124 (7.4)
St Louis	138 (8.2)
Psychiatric diagnosis	991 (58.9)
Hospital admission	712 (42.3)

^a^ Other race includes American Indian or Alaska Native, Asian, Native Hawaiian or Pacific Islander, and mixed or any other patient self-reported race.

### Outpatient Referral at Discharge—Primary Outcome

Overall, 299 of 1683 patients (17.8%) received a referral for outpatient follow-up at the time of discharge. In the unadjusted analysis, patients with psychiatric diagnoses (193 patients with a psychiatric diagnosis [12.3%] vs 106 patients with no psychiatric diagnosis [6.7%]; *P* = .02) or hospital admissions (176 with hospital admission [11.2%] vs 123 without hospital admission [7.8%]; *P* < .001) were significantly more likely to receive a referral for outpatient treatment at discharge. Study site was also significantly associated with differences in rates of discharge outpatient referral. All referral rates for each individual site are reported in Table 2.

**Table 2.** Analysis of Outpatient Follow-Up Referral at Discharge

Variable	Referral, No. (%)		aOR (95% CI)
	Outpatient (n = 299)	No outpatient (n = 1275)	
Age, mean (SD)	42.6 (14.3)	42.6 (14.5)	0.99 (0.98-1.00)
Gender			
Male	222 (14.1)	923 (58.7)	1 [Reference]
Female	77 (4.9)	351 (22.3)	0.85 (0.62-1.18)
Race			
Black^a^	89 (5.7)	328 (20.8)	0.67 (0.47-0.97)
Unknown	46 (2.9)	212 (13.5)	0.84 (0.47-1.48)
White	151 (9.6)	660 (41.9)	1 [Reference]
Other^b^	13 (0.8)	75 (4.8)	0.65 (0.33-1.28)
Ethnicity			
Hispanic	63 (4.3)	313 (21.3)	0.74 (0.48-1.14)
Non-Hispanic	219 (14.9)	874 (59.5)	1 [Reference]
Site^c^			
Atlanta	23 (1.4)	57 (3.6)	1 [Reference]
Bethlehem^a^	14 (0.9)	146 (9.3)	0.26 (0.12-0.57)
Denver^a^	36 (2.3)	200 (12.7)	0.47 (0.25-0.92)
Grand Rapids^a^	11 (0.7)	96 (6.1)	0.34 (0.15-0.77)
Los Angeles	13 (0.8)	58 (3.7)	0.61 (0.26-1.40)
New York	96 (6.1)	368 (23.4)	1.14 (0.61-2.12)
Newark	24 (1.5)	41 (2.6)	1.35 (0.61-3.00)
Pittsburgh	35 (2.2)	104 (6.6)	1.21 (0.61-2.40)
Portland^a^	7 (0.4)	112 (7.1)	0.22 (0.09-0.57)
St Louis	40 (2.5)	93 (5.9)	1.63 (0.84-3.16)
Psychiatric history^c^			
Yes	193 (12.3)	725 (46.1)	1.22 (0.91-1.65)
No	106 (6.7)	550 (34.9)	1 [Reference]
Hospital admission^c^			
Admitted^a^	176 (11.2)	450 (28.6)	3.13 (2.34-4.20)
Not admitted	123 (7.8)	825 (52.4)	1 [Reference]

Abbreviation: aOR, adjusted odds ratio.

^a^ Covariate significantly associated with outcome on adjusted analysis.

^b^ Other race includes American Indian or Alaska Native, Asian, Native Hawaiian or Pacific Islander, and mixed or any other patient self-reported race.

^c^ *P* < .05 on unadjusted analysis.

Following adjustment for demographic and clinical covariates, Black patients had significantly lower adjusted odds ratio (aOR) of receiving an outpatient referral at discharge when compared with White patients (aOR, 0.67; 95% CI, 0.47-0.97). Hospital admission remained associated with increased adjusted odds of receiving an outpatient referral at discharge (aOR, 3.13; 95% CI, 2.34-4.20). Four study sites (ie, Bethlehem, Denver, Grand Rapids and Portland) were associated with decreased adjusted odds of outpatient referral. Full results are provided in Table 2.

### Naloxone Kit or Prescription at Discharge—Secondary Outcome

A total of 713 patients (42.4%) received a naloxone kit or a naloxone prescription at discharge. On unadjusted analysis, younger patients were significantly more likely to receive a naloxone kit or prescription. Patients with prior psychiatric history (379 patients with psychiatric history [23.6%] vs 334 patients without psychiatric history [20.8%]; *P* < .001) and those not admitted to the hospital (393 with hospital admission [24.5%] vs 320 without hospital admission [20.0%]; *P* = .004) were significantly more likely to receive a naloxone kit or prescription at discharge. Race was also significantly associated with differences in rates of naloxone dispensing at discharge, with Black patients being less likely to receive naloxone (168 did not receive naloxone [16.3%] vs 221 received naloxone [10.5%]; *P* < .001). Clinical site was also significantly associated with differences in naloxone dispensing at discharge. Full results are provided in Table 3.

**Table 3.** Analysis of Naloxone Kit or Prescription at Discharge

Variable	Kit or prescription, No. (%)		aOR (95% CI)
	Naloxone (n = 713)	No Naloxone (n = 891)	
Age, mean (SD)^a^	41.4 (13.9)	43.6 (15)	1.00 (0.99-1.01)
Sex			
Male	504 (31.4)	659 (41.1)	1 [Reference]
Female	209 (13)	231 (14.4)	1.11 (0.90-1.49)
Race^a^			
Black	168 (10.5)	261 (16.3)	0.91 (0.68-1.23)
Unknown	76 (4.7)	175 (10.9)	0.87 (0.54-1.39)
White	427 (26.6)	406 (25.3)	1 [Reference]
Other^b^	42 (2.6)	49 (3.1)	1.05 (0.63-1.74)
Ethnicity			
Hispanic	167 (11.2)	210 (14)	1.24 (0.89-1.72)
Non-Hispanic	505 (33.7)	616 (41.1)	1 [Reference]
Site^a^			
Atlanta	49 (3.1)	30 (1.9)	1 [Reference]
Bethlehem	95 (5.9)	66 (4.1)	0.83 (0.45-1.52)
Denver	168 (10.5)	75 (4.7)	1.30 (0.73-2.31)
Grand Rapids	74 (4.6)	38 (2.4)	1.15 (0.61-2.17)
Los Angeles	43 (2.7)	31 (1.9)	0.77 (0.38-1.54)
New York^c^	114 (7.1)	346 (21.6)	0.20 (0.11-0.35)
Newark^c^	16 (1)	57 (3.6)	0.14 (0.06-0.32)
Pittsburgh^c^	40 (2.5)	109 (6.8)	0.24 (0.13-0.45)
Portland^c^	58 (3.6)	64 (4)	0.50 (0.27-0.95)
St Louis^c^	56 (3.5)	75 (4.7)	0.46 (0.25-0.83)
Psychiatric history^a^			
Yes	379 (23.6)	553 (34.5)	0.93 (0.74-1.17)
No	334 (20.8)	338 (21.1)	1 [Reference]
Hospital admission^a^			
Admitted	320 (20.0)	337 (21.0)	1.11 (0.88-1.40)
Not admitted	393 (24.5)	554 (34.5)	1 [Reference]

Abbreviation: aOR, adjusted odds ratio.

^a^ *P* < .05 on unadjusted analysis.

^b^ Other race includes American Indian or Alaska Native, Asian, Native Hawaiian or Pacific Islander, and mixed or any other patient self-reported race.

^c^ Covariate significantly associated with outcome on adjusted analysis.

Following adjustment for demographic and clinical covariates, 5 study sites (ie, New York, Newark, Pittsburgh, Portland, and St. Louis) were associated with decreased adjusted odds of naloxone dispensing at discharge. Full results are provided in Table 3.

### Buprenorphine Prescription at Discharge—Secondary Outcome

A total of 141 patients (8.4%) received a prescription for buprenorphine at discharge. On unadjusted analysis, Hispanic patients were significantly less likely to receive buprenorphine (22 Hispanic patients [1.5%] vs 111 non-Hispanic patients [7.3%]; *P* = .02), although there was no difference once adjusted for clinical site location, demographics, and clinical history. Patients with hospital admission (111 patients who were admitted [6.8%] vs 30 patients were not admitted [1.8%]; *P* < .001) were also significantly more likely to receive buprenorphine at discharge. Race was associated with significant differences in rates of buprenorphine prescription at discharge. For example, 761 White patients (46.7%) did not receive buprenorphine vs 80 White patients (4.9%) who received buprenorphine and 393 Black patients (24.1%) did not receive buprenorphine vs 44 Black patients (2.7%) who received buprenorphine (*P* = .04). Study site was also associated with significant differences in rates of buprenorphine prescription at discharge. Full results are provided in Table 4.

**Table 4.** Analysis of Buprenorphine Prescription at Discharge

Variable	Prescription, No. (%)		aOR (95% CI)
	Buprenorphine (n = 141)	No buprenorphine (n = 1489)	
Age, mean (SD)	43.1 (14.2)	42.5 (14.5)	1.00 (0.99-1.02)
Sex			
Male	100 (6.1)	1086 (66.7)	1 [Reference]
Female	41 (2.5)	402 (24.7)	1.13 (0.74-1.73)
Race^a^			
Black	44 (2.7)	393 (24.1)	0.61 (0.37-1.02)
Unknown	12 (0.7)	248 (15.2)	0.91 (0.35-2.34)
White	80 (4.9)	761 (46.7)	1 [Reference]
Other^b^	5 (0.3)	87 (5.3)	0.66 (0.25-1.79)
Ethnicity^a^			
Hispanic	22 (1.5)	361 (23.8)	0.65 (0.36-1.18)
Non-Hispanic	111 (7.3)	1025 (67.5)	1 [Reference]
Site^a^			
Atlanta	20 (1.2)	62 (3.8)	1 [Reference]
Bethlehem	24 (1.5)	137 (8.4)	0.72 (0.33-1.57)
Denver^c^	19 (1.2)	225 (13.8)	0.31 (0.14-0.67)
Grand Rapids^c^	4 (0.3)	108 (6.6)	0.15 (0.05-0.49)
Los Angeles^c^	4 (0.3)	70 (4.3)	0.21 (0.06-0.70)
New York^c^	19 (1.2)	450 (27.6)	0.22 (0.09-0.50)
Newark	17 (1)	61 (3.7)	1.07 (0.47-2.45)
Pittsburgh^c^	7 (0.4)	144 (8.8)	0.24 (0.09-0.64)
Portland	8 (0.5)	114 (7)	0.38 (0.15-1.00)
St Louis	19 (1.2)	118 (7.2)	0.93 (0.43-1.99)
Psychiatric history			
Psychiatric history	85 (5.2)	868 (53.3)	1.20 (0.80-1.79)
No psychiatric history	56 (3.4)	621 (38.1)	1 [Reference]
Hospital admission^a^			
Admitted^c^	111 (6.8)	556 (34.1)	5.84 (3.71-9.19)
Not admitted	30 (1.8)	933 (57.2)	1 [Reference]

Abbreviation: aOR, adjusted odds ratio.

^a^ *P* < .05 on unadjusted analysis.

^b^ Other race includes American Indian or Alaska Native, Asian, Native Hawaiian or Pacific Islander, and mixed or any other patient self-reported race.

^c^ Covariate significantly associated with outcome on adjusted analysis.

Following adjustment for demographic and clinical covariates, hospital admission remained associated with increased adjusted odds of receiving a buprenorphine prescription at discharge (aOR, 5.84; 95% CI, 3.71-9.19). Full results are provided in Table 4.

## Discussion

In this national study of 1683 patients presenting to the hospital for a suspected acute opioid overdose, we observed racial disparities in outpatient referrals for OUD and overall low rates of OUD treatment referrals and prescription or provision of buprenorphine and naloxone at discharge. We also noted variations in referral and treatment by study site, presence of prescription opioids or fentanyl on toxicologic analysis, and hospital admission status. Racial disparities in ED care were particularly disconcerting, considering that the ED serves as an important health care safety net; at times, the ED represents the only accessible medical care for a subset of some racial minority and vulnerable socioeconomic groups.^18,19^ These data were consistent with, and build upon, prior studies demonstrating that Black ED patients were less likely to receive buprenorphine^7^ or antidotal therapy^20^ as compared with White ED patients. Prior data from the 2022 National Survey on Drug Use and Health similarly found that Black patients were less likely to receive buprenorphine than White patients; unlike the present study, this prior national survey also noted disparities in buprenorphine receipt among female patients, Hispanic patients, and patients at extremes of age. This variability may be due to the fact that the scope of the present analysis was focused solely on ED patients presenting with suspected opioid overdose (ie, did not include patients with OUD who presented with withdrawal or other medical concerns), geographic variation in patient demographics, and sample size limitations in the present study.^1^ Nonetheless, findings from the present study underscore the need for targeted interventions to address racial disparities in ED care for OUD, particularly in enhancing referral processes and buprenorphine prescribing practices for Black patients.

Patients who were admitted to the hospital were more likely to receive referrals to treatment and a buprenorphine prescription, potentially reflecting a perception that these patients suffered a more severe overdose, and, therefore, have a bigger need for intervention. It is also possible that this is reflective of the longer window for health care intervention. Patients admitted to the hospital may have also been more likely to experience opioid withdrawal during the course of their hospitalization, which may in turn have led to increased rates of buprenorphine prescription and outpatient treatment linkage. Due to the increased length of stay, admitted patients may have also been more likely to be engaged by social work, care navigators, and other ancillary clinicians.

Although we observed a much higher rate of naloxone dispensing or prescription than was reported in prior studies,^12,13^ this may be due to differences in methods. Notably, both cited studies used claims records reflecting filled prescriptions. In prior observational studies, fewer than 1 in 5 naloxone prescriptions provided from the ED were filled.^21,22^ The reasons for this are multifactorial and can include financial barriers, lack of transportation, and stigma, among others, and must continue to be addressed. All sites in this study were able to either provide a naloxone kit or a prescription for naloxone at the time of discharge. For this study, we were unable to determine if naloxone prescriptions were filled, and did not collect information on whether naloxone was prescribed or dispensed as a naloxone kit at the time of discharge.

There was wide variability in referrals, naloxone dispensing or prescription, and buprenorphine prescribing across study sites, likely reflecting the diversity of practice settings and resources available at each site. It is likely that sites had differential access to follow up care, including clinics that prescribe medications for OUD. While this dataset highlights significant disparities, it does not allow us to draw any conclusions about whether conscious or unconscious health care professional bias is driving referral and treatment rates based on race or ethnicity. Existing literature on health care disparities often attributes such differences to unconscious bias rather than deliberate actions. Nonetheless, further research is necessary to validate and expand upon these critical findings. Furthermore, we did not collect information about patients’ prior engagement in treatment, and it is possible that patients did not receive a referral for ongoing treatment or medication prescription because they were already engaged with these resources prior to their overdose. We also did not collect data on whether a lack of treatment referral, naloxone dispensing or prescribing and buprenorphine prescription was due to clinician factors (ie, clinician not offering referral or naloxone) or patient factors (patient refusal of referral, naloxone kit or buprenorphine). Medical mistrust and other patient-level factors may disproportionately impact treatment acceptance and engagement among Black patients.^23,24^ As such, additional research is needed to further identify, explore, and address specific clinician and patient-level factors in disparities in OUD treatment across regions and practice settings.

The present study is consistent with prior findings from a similar ED patient population. Previously, Black and Hispanic patients were found to be significantly less likely to receive any antidote when presenting to the ED for acute overdose from any drug.^20^ It is not clear exactly how or why race and ethnicity may drive OUD treatment interventions, although one might speculate that the observed disparity may be clinician driven and implicit bias may be a contributing factor.^25^ Additional studies exploring race, ethnicity, and antidote administration in other cohorts with greater clinical detail are necessary to determine the national prevalence of disparities in the management after an acute overdose in the ED and the potential impact of any such disparities on patient outcomes. Additionally, assessment of possible additional factors, such as insurance status, clinician race and ethnicity, and hospital level barriers must also be explored in future research. As health care delivery models evolve, it is imperative to ensure that patients of all racial and ethnic backgrounds receive equitable, high-quality care at every access point within the health care system, including the ED.

### Limitations

This study has limitations. Given its methods and the self-reported nature of some study variables including patient demographic variables, data in the medical record may have been incomplete or inaccurate, and errors could have been introduced during the data extraction process. Dedicated TOXIC staff follow up with participating sites if incomplete or mismatching data were found during the ongoing quality assurance process.

This study was subject to limitations due to inclusion criteria for the parent Fentalog study. Patients were included in the Fentalog study if they presented to the ED with a suspected opioid overdose. Accordingly, patients who presented to the ED for overdose presentations not suggestive of a primary opioid toxidrome were not captured in this analysis. This may have limited our full ability to capture patients with adulterated opioid exposure in the present analysis. Patients were also included only if they had waste blood available for analysis. This may have biased the included sample of patients toward those with a more severe overdose presentation as patients with more severe overdose are more likely to have blood testing done as part of routine clinical care.

Our study examined buprenorphine prescription at discharge as an outcome measure reflective of ED initiation of buprenorphine for opioid use disorder. We did not report or analyze in-ED administration of buprenorphine because reasons for in-ED administration were variable (eg, some patients were given buprenorphine in the ED because they missed their standing home dose) and were not reflective of ED opioid use disorder treatment initiation. However, this may have led to our analysis not capturing patients who were given high-dose ED buprenorphine or long-acting injectable formulations of buprenorphine and discharged without a prescription. Many EDs also now discharge patients with buprenorphine to-go or starter packs, and there may be variability in whether these packs require a conventional prescription. Additionally, we did not collect data about ED patients who were initiated on methadone for opioid use disorder. Nationally, buprenorphine is the most common ED-initiated medication for opioid use disorder and has fewer regulatory barriers to dispensing and prescribing from the ED setting.^26^ However, depending on outpatient treatment access and local practice patterns, some EDs may preferentially use methadone for patients with opioid use disorder. As such, it is possible that some patients within our analysis who did not receive buprenorphine prescriptions instead were given methadone and were thus erroneously considered to have not received ED treatment or referral. Prior studies have noted that Black patients with OUD are also more likely to receive methadone for treatment when compared with White patients^27^; accordingly, if a disproportionate number of Black patients in the present study were given methadone or were already on established methadone treatment, this may have contributed to observed disparities in treatment referral rates.

Additionally, we did not review pharmacy records and were unable to determine if prescriptions for buprenorphine or naloxone were filled. We also did not perform a follow-up analysis to determine if patients who were linked to outpatient appointments ultimately attended these follow-up appointments. Furthermore, we had a relatively small sample size of patients who received buprenorphine prescriptions and outpatient treatment referrals, limiting multivariable analysis for these study outcomes. The Fentalog Study data also lacks granularity about reasons naloxone was not dispensed or prescribed at discharge. In some prior studies, nearly 50% of patients who were offered naloxone on discharge declined and patients who refused naloxone cited varied reasons for this refusal.^28,29^ Accordingly, it is possible that a substantial proportion of patients in our study who did not receive naloxone at discharge were offered naloxone but refused.

Additionally, it is possible that some of the associations identified in our analysis may be affected by individual site variability regarding access to outpatient referral and racial or ethnic makeup of the site’s patient population. While our analysis adjusted for clinical site in multivariable analysis, a more in-depth analysis of variation due to individual site and regional care access and demographics is outside the scope of this analysis.

Finally, toxicologic testing was qualitative in nature. Positive results represent recent exposure and do not necessarily confirm that all of the substances detected caused or contributed to the patient’s acute overdose presentation.

## Conclusions

In this large multicenter national cohort of patients with suspected opioid overdose, Black patients were less likely to receive outpatient referrals for OUD. Overall, provision of treatment referral, naloxone kits or prescriptions, and buprenorphine prescription at discharge were low, ranging from 8% to 44%, suggesting significant room for improvement in ED harm-reduction practices. Admitted patients were also more likely to receive outpatient referrals and buprenorphine than those discharged from the ED. These findings underscore the need for targeted interventions to address racial disparities in ED care for OUD, particularly in enhancing referral processes and buprenorphine prescribing practices for Black patients.

## Notes

Supplement.
Data Sharing Statement
